# Local reference dose evaluation in conventional radiography examinations in Iran

**DOI:** 10.1120/jacmp.v15i2.4550

**Published:** 2014-03-06

**Authors:** M. Shirin Shandiz, M.T. Bahreyni Toossi, S. Farsi, K. Yaghobi

**Affiliations:** ^1^ Department of Medical Physics Zahedan University of Medical Sciences Zahedan Iran; ^2^ Department of Medical Physics and Biomedical Engineering Tehran University of Medical Sciences Tehran Iran; ^3^ Medical Physics Research Centre, Faculty of Medicine Mashhad University of Medical Sciences Mashhad Iran; ^4^ Department of Occupational Health Iran University of Medical Sciences Tehran Iran

**Keywords:** conventional radiography, dose area product, exposure factors, radiation protection, reference dose

## Abstract

The goal of this study was to establish local diagnostic reference levels (LDRLs) for conventional radiography examinations in Sistan‐Baluchestan province of Iran, using dose area product (DAP) measurements followed by a comparison with international dose levels. DAP factor evaluation was carried out at eight radiography rooms in six public and one private health‐care centers. The study employed DAP, exposure, and demographic data (weight, age, height) for 1069 patients who presented for one of the 11 routine radiography examinations: chest (AP, PA, LAT), abdomen (AP), lumbar spine (AP, LAT), pelvis (AP), skull (AP/PA, LAT), and cervical spine (AP, LAT). The data were analyzed statistically and the minimum, median, mean, maximum, and third quartile DAP values were calculated. It was observed that LDRLs for chest PA (0.26 Gy.cm^2^) and chest LAT (0.66 Gy.cm^2^) projections were up to 136% and 113% higher, respectively, than their corresponding NRPB 2005 values. Other radiographic procedures had lower recommended reference doses compared with recently recommended national reference doses published in recent NRPB reports and other studies. Wide variations in DAP values and exposure parameters were observed for similar radiographic procedures between patients in different rooms and for different patients in the same room. These and other observations, such as poor radiographic techniques, high rate of radiographic reject/repeat, and lack of modern X‐ray machines and equipment, show that the need to carry out quality assurance programs is critical in Iran.

PACS numbers: 87.53.Bn, 87.59.B

## I. INTRODUCTION

NCRP 160 Report states that medical radiation exposure of the United States population is almost half of total radiation exposure from natural and artificial sources.^(1)^ In its 2010 Report, the United Nations Scientific Committee on the Effects of Atomic Radiation (UNSCEAR) indicates that medical radiology is the largest man‐made source of radiation exposure.^(2)^ This is a result of the growing use of diagnostic imaging methods, particularly computed tomography (CT). CT and conventional radiology are the most frequently used and account for most of the cumulative dose from diagnostic radiology methods and, hence, a key topics in radiation protection.^(3)^

The International Commission on Radiological Protection (ICRP) emphasizes three fundamental principles for protection in radiation diagnostic radiology. These are justification, protection optimization, and application of dose and risk limits.^(4)^ The British National Radiological Protection Board (NRPB) emphasizes regular patient dose measurement in all radiological departments and diagnostic reference levels (DRLs) to optimize patient protection. To establish patient DRLs for various radiography tests and raise public awareness about patient dose, it is useful to identify those centers associated with higher radiation doses. Following from this, adopting measures such as quality control of equipment can lead to a reduction in patient doses while maintaining image quality.^(5)^

The last 50 years of dosimetry in the United States have shown that the regular use of quality control programs for diagnostic radiology equipment and the establishment of DRLs by the National Evaluation of X‐ray Trends (NEXT) have played vital roles in reducing patient radiation doses.^(6)^ For example, based on NEXT patient average entrance skin dose (ESD) and data such as reference dose levels, a 50%‐70% reduction in average ESD was achieved for the years 1964 to 2004 for chest PA, abdomen AP, and lumbar‐sacral spine AP radiography examinations.^(6)^ Similar efforts have led to large reductions in patient doses in countries such as the UK. NRPB 2005 (HPA‐RPD‐029) reports that 20 years of regular patient dose monitoring has reduced DRLs by more than 50%.^(7)^

Radiological DRL values are explained in terms of ESD and dose area product (DAP).^(7)^ DAP is a product of the absorbed dose multiplied by the irradiated area. DAP is not only a quick and simple measurement, but also a valuable radiation dose descriptor. Its advantage is that the biological effects of radiation are dependent on radiation dose and the irradiated area of the body. DAP is also applicable for quality assurance and functional analysis of X‐ray machines.^8^, ^9^, ^10^

The United Nations Scientific Committee on the Effects of Atomic Radiation (UNSCEAR 2000) reports that similar examinations in different countries and different districts of the same country may have different values stemming from cultural, scientific, and practical differences between regions. As a result, DRLs can be separately determined for a city, geographical area, or large health‐care centers as local diagnostic reference levels (LDRLs), while nationwide surveys establish national diagnostic reference levels (NDRLs).^(11)^

This study examines the patient dose information and LDRLs in Sistan‐Baluchestan, Iran. LDRLs for common radiography procedures and with DAP measurements were recorded in Sistan‐Baluchestan province, a comparatively disadvantaged and less‐developed province in Iran.

## II. MATERIALS AND METHODS

This study followed guidelines established by NRPB 2005 (HPA‐RPD‐029)^(7)^ and was conducted in eight radiography rooms in six public hospitals and one diagnostic center located in Sistan‐Baluchestan province. They were selected using the random method from 33 functional rooms located in 27 state‐owned and private radiography centers, and formed a reasonable geographical distribution and good distribution of hospital/center sizes.

The province is comparatively disadvantaged and underdeveloped; at the time of the project, there were no computed radiography (CR) and direct digital system in use. The radiographic devices used were one single‐phase, three 3‐phase, and four high‐frequency devices. All radiographic devices used the film‐screen system with a speed of 400 in all rooms. None of the devices had an automatic exposure control (AEC).

Table 1 shows the results for the following diagnostic centers: Private Diagnostic Center (room 1), Khatam Educational, Research and Treatment Center of Zahedan (room 2), Private Diagnostic Center (room 3), Khatam Educational and Treatment Center of Iranshahr (room 4), Social Security Organization Hospital of Zahedan (room 5), Emam Ali Educational, Research and Treatment Center of Zabol (room 6), Bu‐Ali Educational and Treatment Center of Zahedan (room 7), and the Emam Ali Educational and Treatment Center of Chabahar (room 8).

**Table 1:** DAP (mean±SE) for Gy.cm^2^ for radiographies from selected radiography rooms

	*Room*							
	*1*	*2*	*3*	*4*	*5*	*6*	*7*	*8*
Grid ratio	12:1	10:1	12:1	10:1	8:1	10:1	10:1	8:1
Filtration (mm Al)	3.3	3.1	3.1	3.8	2.5	3.2	3.1	3.1
DAP values (mGy.cm^2^) of								
Chest AP	0.37±0.02	0.31±0.06	0.16±0.05		0.19±0.07	0.21±0.10		0.25±0.13
Chest PA	0.26±0.05	0.25±0.09	0.26±0.12	0.21±0.10	0.26±0.33	0.18±0.04	0.10±0.04	0.25±0.05
Chest LAT			0.96±0.19	0.40±0.21	0.37±0.08		0.25±0.09	
Abdomen AP	1.63±0.41	0.84±0.20	2.16±0.79	0.87±0.36	1.65±0.66	0.91±0.27	1.11±0.38	0.44±0.14
Lspine AP	0.88±0.28	0.65±0.21	1.14±0.33	0.48±0.12	1.05±0.37	0.40±0.21	0.99±0.42	0.61±0.17
Lspine LAT	1.63±0.72	0.97±0.35	2.10±0.83	1.26±0.47	2.33±0.90	1.00±0.51	1.79±0.63	1.32±0.34
Pelvis AP	1.64±0.49	0.53±0.21	2.04±0.58	0.44±0.22	1.52±0.81	0.46±0.17	1.19±0.21	1.18±0.79
Skull AP/PA	0.54±0.20	0.27±0.09	0.45±0.21	0.54±0.10	0.27±0.16	0.18±0.03	0.61±0.22	0.42±0.25
Skull LAT	0.40±0.20	0.27±0.07	0.43±0.27	0.47±0.26	0.22±0.12		0.46±0.17	0.39±0.06
Cervical S AP	0.57±0.14	0.25±0.06	0.13±0.21		0.18±0.19		0.34±0.09	
Cervical S LAT	0.15±0.07	0.18±0.32	0.74±0.51		0.37±0.14		0.21±0.08	

SE=standard error.

A DAP meter (Gammex RMI, Model 840A; Middleton, WI), calibrated according to the method proposed by NRPB protocol, was used to obtain DAP values.^(9)^ The system had a detector and a monitor. The detector was 14 cm×14 cm and was installed under the beam collimator. It had a diagnostic energy range of 50 to 150 kVp and low absorption (less than 0.5 mm Al).

An initial quality control test (timer accuracy, kVp accuracy, mA linearity, mAs reciprocity, HVL check, and output check) was performed in all rooms on the facilities using a Mult‐O‐Meter model 303 produced by UNFORS, Sweden. This is a multiparameter X‐ray meter with an internal silicon detector which can measure kVp, dose, rate, and time with maximum inaccuracy of 0.5% for measuring the time and 2% for others. For each X‐ray machine, the measured HVL, the tube wave‐form and anode angle for 80 kVp are used for total filtration estimation.^12^, ^13^

After patient exposure, DAP values (mGy.cm^2^) and radiation time (ms) were transmitted via cable to the monitor. This and other radiographic data (kVp, mA, mAs), radiography conditions (filtration, grid ratio), and patient information (gender, age, height, weight, BMI) were recorded. Body mass index (BMI), derived from weight/height^2^ (kg/m^2^), is a useful classification scheme for the size and shape of a person.^(14)^ Since patient dose depends on patient size,^(7)^ information was collected for adult patients over age 16 weighing 45 to 120 kg and having a BMI 14 to 40.

Average DAP values were calculated from the measurements for each room for the 11 conventional examinations considered in this study: chest (AP, PA, LAT), abdomen (AP), lumbar spine (AP, LAT), pelvis (AP), and skull (AP/PA, LAT). The third quartile DAP values were then calculated from the results for each radiographic examination type and view and adopted as the LDRL in Sistan‐Baluchestan province.^(7)^

## III. RESULTS

Average DAP values (Gy.cm^2^), total filtration (mm Al) at 80 kVp, and grid ratio for X‐ray machines for the studied rooms are shown in Table 1. Only one grid ratio was used in each X‐ray machine for Bucky stand and Bucky table. Table 2 lists the radiological parameters (examination type, kVp, mAs) and the spread of the 1069 patients across the radiographic examinations. The age, gender, weight, and BMI of the patients are also presented in Table 2.

Table 3 shows the statistical distribution of the average DAP by room and the minimum, maximum, mean, median, first quartile, third quartile, and maximum‐to‐minimum ratio for the 11 radiographic procedures. Statistical distributions for all radiographs were obtained; the chest AP had the minimum sample size in this study.

Table 4 shows the average dose (Gy.cm^2^) for other studies for comparison purposes. Studies conducted by Bahreyni Toossi et al. in 2006 in the city of Mashhad and in 2011 in the city of Sabzovar are the only studies in Iran that used DAP. Table 4 presents results for NRPB 2000 (W14) and NRPB 2005 (HPA‐RPD‐029) for average dose and UNSCEAR 2000 recorded for nationwide patient dose evaluations conducted in Germany, New Zealand, and Finland. Also included are the results of a study by Akinlade et al. conducted on a limited scale in four hospitals in Nigeria.^7^, ^10^, ^11^, ^15^, ^16^, ^17^

Table 5 includes kVp and mAs values which are routinely used for different types of radiographic examinations in different places. Since there is a shortage of DAP studies, a review of studies where ESDs were measured by Kim et al.^(18)^ in South Korea, Sonawane et al.^(19)^ in

**Table 2:** Average patient characteristics and exposure parameters. The range from minimum to maximum for individual patients is given in brackets

*Radiograph*	*Number*	*Male / Female Ratio*	*Patients' Weight (kg)*	*BMI*	*Patients' Age*	*Tube Voltage (kVp)*	*mAs*
Chest AP	21	9/12	64(45−101)	26(14−40)	36(16−80)	64(56−78)	20(4−32)
Chest PA	258	125/133	65(45−110)	24(13−40)	46(16−89)	65(50−87)	17(4−128)
Chest LAT	36	16/20	66(45−86)	23(18−24)	50(16−82)	70(62−74)	29(12−37)
Abdomen AP	73	39/34	67(45−95)	25(14−37)	42(16−82)	69(50−110)	43(16−80)
Lspine AP	152	70/82	68(45−109)	26(17−38)	40(16−75)	70(55−93)	53(6−256)
Lspine LAT	150	70/80	67(45−100)	25(16−38)	38(16−82)	80(59−104)	73(8−256)
Pelvis AP	76	48/28	69(45−105)	25(15−40)	38(16−85)	65(52−79)	38(4−96)
Skull AP/PA	83	43/40	67(45−104)	24(14−32)	29(16−90)	65(48−85)	36(3−96)
Skull LAT	49	30/19	68(45−104)	24(14−35)	31(16−90)	60(49−86)	26(7−60)
Cervical S AP	75	20/55	67(45−120)	25(16−40)	40(17−78)	60(51−80)	21(4−64)
Cervical S LAT	89	32/57	67(46−120)	25(14−40)	41(16−84)	61(48−81)	20(3−64)
All	1062	502/560	66(45−120)	25(14−40)	40(16−90)	67(48−110)	35(3−256)

**Table 3:** Statistical distribution of average DAP values per room

	*Number of*									
*Radiograph*	*Centers*	*Rooms*	*Patients*	*mean*	*Min.*	*Max.*	*First Quartile(1st)*	*Median*	*Third Quartile(3rd)*	*Max/Min*
Chest AP	4	4	21	0.25	0.08	0.64	0.18	0.24	0.33	8
Chest PA	7	8	258	0.22	0.03	0.7	0.19	0.24	0.26	23
Chest LAT	5	6	36	0.5	0.19	1.14	0.29	0.39	0.66	6
Abdomen AP	7	8	73	1.29	0.42	3.43	0.91	1.14	1.64	8.16
Lspine AP	7	8	152	0.7	0.1	3.21	0.43	0.61	1.02	32
Lspine LAT	7	8	150	1.52	0.17	5.92	1.07	1.48	1.97	35
Pelvis AP	7	8	76	1.09	0.09	2.94	0.44	0.9	1.64	33
Skull AP/PA	7	8	83	0.42	0.02	1.4	0.28	0.42	0.59	70
Skull LAT	6	7	49	0.39	0.02	1.38	0.27	0.43	0.46	69
Cervical S AP	4	5	75	0.15	0.05	0.87	0.09	0.14	0.16	17.4
Cervical S LAT	4	5	89	0.16	0.04	1.25	0.1	0.14	0.25	31.25

**Table 4:** Average DAP for present study versus other studies

	*Bahreyni Toossi et al, Iran*						*UNSCEAR 2000*		
*Radiograph*	*This Study*	*Mashad City 2006*	*Sabzevar City 20ll*	*NRPB 2005*	*NRPB 2000*	*Bidemi 2012*	*Finland*	*New Zealand*	*Germany*
Chest AP	0.252	0.629	0.434	0.11					
Chest PA	0.219	0.578	0.258	0.09	0.1	1.25^a^	0.44	0.17	1.37^d^
Chest LAT	0.5			0.25		1.25^a^		0.62	1.37^d^
Abdomen AP	1.285	1.881	0.852	2.16	2.5	0.56	6.9	2.67	3.62
Lspine AP	0.709	2.699	0.502	1.33	1.4		8.3	1.88	9.32^e^
Lspine LAT	1.515	2.34	1.085	2.14	2.3			3.92	9.32^e^
Pelvis AP	1.105	2.076	0.744	1.9	2.2	0.464	3.8	2.37	3.62
Skull AP/PA	0.424	1.176		0.62		0.340^b^	1.6	0.96	1.07^f^
Skull LAT	0.386	0.778		0.51		0.340^b^		0.57	1.07^f^
Cervical S AP	0.151	0.341	0.128			0.266^c^			
Cervical S LAT	0.158	0.351	0.165			0.266^c^			

^a, b, c, d, e, f^ Values are reported together.

**Table 5:** Conventional average radiation parameters (kVp, mAs) from different studies

					*Bahreyni Toossi et al. Iran*						
*Nigeria 2012 DAP*	*India 2010 ESD*	*Korea 2007 ESD*	*NRPB 2005 DAP*	*NRPB 2000 DAP*	*Tehran ESD*	*Sabzovar DAP*	*Mashhad DAP*	*This Study DAP*	*Parameter*	*Projection*	*Radiograph*
80	67	74	73	73	68	70	80	69	kVp	AP	Abdomen
65	67	33	93	54	54	30	58	43	mAs		
	61		73		61	66	83	64	kVp	AP	
	15		17		22	30	31	20	mAs		
85	60	106	84	83	66	68	122	65	kVp	PA	Chest
	17	9	4	5	18	18	16	17	mAs		
	66	104	88		72			70	kVp	LAT	
	32	25	11		41			29	mAs		
	69	76	76	76	70	72	91	70	kVp	AP	Lspine
	80	35	43	50	50	44	72	53	mAs		
	78	84	87	87	80	78	100	80	kVp	LAT	
	118	68	54	64	73	68	84	73	mAs		
78	68	72	71	74	66	67	82	65	kVp	AP	Pelvis
77	69	31	193	46	48	40	61	38	mAs		
72	68	72	68		64	63	74	65	kVp	AP/PA	Skull
36	66	28	20		42	34	26	36	mAs		
72	65	69	65		59	59	68	60	kVp	LAT	
36	60	25	16		32	29	18	26	mAs		
		68			61	61	74	60	kVp	AP	Cervical S
		19			28	17	35	21	mAs		
		74			59	63	79	61	kVp	LAT	
		25			21	17	36	20	mAs		

India, and Bahreyni Toossi and Asadinezhad^(20)^ in Tehran was used to obtain comparable data. Table 6 shows DRLs for different types of radiographic examinations in Sistan‐Baluchestan province and recent NRPB reports.

**Table 6:** DRLs from present survey vs. recommended national reference doses from recent NRPB reports

	*Diagnostic Reference Levels (Gy. cm^2^)*		
*Radiograph*	*This study*	*NRPB2000*	*NRPB2005*
Chest AP	–	–	–
Chest PA	0.26	0.12	0.11
Chest LAT	0.66	–	0.31
Abdomen AP	1.64	3	2.6
Lspine AP	1.02	1.6	1.6
Lspine LAT	1.97	3	2.5
Pelvis AP	1.64	3	2.1
Skull AP/PA	0.59	–	0.78
Skull LAT	0.46	–	0.49
Cervical S AP	0.16	–	–
Cervical S LAT	0.25	–	–

## IV. DISCUSSION & CONCLUSION

1, 2, 3 show a wide range of DAP values and exposure parameters for similar radiographic procedures. These variations were also observed in one room, for a specific procedure for different patients and in different rooms for similar procedures. For instance, the maximum‐to‐minimum ratio of DAP for individual patients varied from 6 for chest LAT to 70 for skull AP/PA. Such wide variations have also been reported elsewhere in diagnostic radiography practice^21^, ^22^, ^23^ and suggest that doses can be reduced without loss in image quality.

Table 4 shows that the average DAP value incurred by patients following chest AP, PA, and LAT projections are higher than corresponding values presented in NRPB 2000 (W14)^(10)^ and 2005 (HPA‐RPD‐029).^(7)^ At the same time, the patient dose in the present study for these examinations are smaller than analogous figures acquired by Bahreyni Toossi et al.,^(1516)^ Akinlade et al.,^(17)^ and similar data obtained by UNSCEAR 2000 in New Zealand and Finland. ^(11)^ A comparison of the studies for other radiographic examination (abdomen (AP), lumbar spine (AP, LAT), pelvis (AP), skull (AP/PA, LAT), and cervical spine (AP, LAT)) based on average DAP values show that results of this study are lower than most other international reports presented in Table 4, but higher than Bahreyni Toossi et al.^(15)^ in Sabzevar that used a similar film screen speed.

NRPB 2000 and The Commission of European Communities (CEC) recommend the use of grid in high kVp (110‐150 kVp) and high FFD (180 cm) and not using grid with low and mid kVp ranges (60‐90 kVp) for chest PA radiographies.^10^, ^24^ Nevertheless, radiologic technologists in this study often (92%) used grid for chest PA radiographies in the low and mid kVp ranges (50‐87 kVp) and low FFD (145 cm±45 cm) in the upright position (1, 2). Lower FFD would be expected to somewhat decrease doses, but Table 5 indicates that the use of grid with low and mid kVp technique is the main reason for higher DAP values for chest radiographies than recommended in NRPB reports.

Nevertheless, the exposure parameters and exposure values have not been approved for the Bucky factor (the ratio of exposure with the grid to exposure without the grid) and were not sufficiently increased (2, 4, 5).^(25)^ The ratio of exposure with the grid to exposure without the grid for chest PA examinations (similar kVp and FFD) are calculated for room 6 and 7. While the Bucky factor is about 3.25 for mid kVp and grid ratio:10 in the references, average of them are about 2.5 for mid kVp and grid ratio:10 in current study.^(25)^ High rate of radiographic reject/repeat observed in this study and previous study that confirmed this results and poor exposure parameters. Our previous study showed that overall percentages of the repetition of radiographic images were 12.9% in Sistan‐Baluchestan province health care centers.^(26)^

It seems that DAP doses that are higher (e.g., chest radiographies) and lower (other radiographic procedures) than corresponding values presented in other reports (Table 5) may be a result of poor radiographic techniques or noncompliance with standard guidelines (such as CEC guidelines about kVp, mAs, FFD, grid ratio, total filtration, collimation, etc.) for good radiographic image in this province.^7^, ^10^, ^11^, ^15^, ^16^, ^17^, ^18^, ^19^, ^20^ As 4, 5 show, this weakness was also seen in Bahreyni Toosi et al. in Tehran, Mashhad, and Sabzovar.^15^, ^16^, ^20^

Table 6 shows similar to average doses for chest PA and LAT. The third quartile DAP values for Sistan‐Baluchestan province that were recommended as the LDRL are higher than NRPB 2005 (HPA‐RPD‐029) by up to 136% and 113%, respectively/^27,7^ There was insufficient data for the chest AP (Table 3; four centers and rooms, 21 patients); therefore, a recommended LDRL was not developed for this procedure.

Since Sistan‐Baluchistan is underdeveloped, all centers in this study used only film‐screen system. Only 55% of centers in the NRPB 2005 (HPA‐RPD‐024) used a film‐screen combination; the rest were equipped with CR (40%) and a direct digital system(5%).^(7)^ Most X‐ray machines were equipped with an AEC system, which may affect the magnitude of DAP and, consequently, DRLs.^(7)^ The radiography technology and film‐screen speed applied in this study (speed class 400) were similar to NRPB 2000 (W14).^(10)^ About 98% of radiography devices use a film‐screen system with an average film screen speed of 390.^(10)^ The LDRL for chest PA radiography is 117% higher than the corresponding NRPB 2000 value, but recommended local reference doses for abdomen AP, lumbar spine AP, lumbar spine LAT, and pelvis AP for Sistan‐Baluchestan province are lower than those in NRPB 2000 by 45%, 36%, 34%, and 45%, respectively (Table 6).^7^, ^10^ These findings also confirm that radiographic techniques in Sistan‐Baluchestan province healthcare centers are poor and inadequate.

DRLs for cervical spine AP and LAT using DAP measurements were not reported in NRPB and other studies.^7^, ^27^ Table 6 shows that the first DRLs for these are 0.16 and 0.25 Gy.cm^2^, respectively.

Average BMI was 25kg/m^2^ and average weight was 66 kg (Table 2); this average weight is less than the 70 kg mean weight in NRPB 2000 and 2005.^7^, ^10^ BMI was not considered in NRPB 2000 and 2005.^7^, ^10^ This factor is a function of weight and height and patients with larger mass require higher technical parameters (kVp and mAs). Average BMI is higher in Sistan‐Baluchestan than analogous values in Iran and European countries;^(28)^ larger BMI values could lead to higher radiation dose and DRLs.^29^, ^30^

In 2005, more than six million chest radiographies were performed, accounting for 30% of the total radiography examinations nationwide (Iran). The number of chest X‐rays was larger and DRL for chest X‐rays were higher in this study than for similar studies.

Observations such as wide range of DAP values and exposure parameters for similar radiographic procedures, poor radiographic techniques, high rate of radiographic reject/repeat, and lack of modern X‐ray machines and equipment (e.g. AEC, DR, and CR detector) are all convincing reasons for a comprehensive QA program. Such a QA program should implement measures such as using the smallest possible radiation field, appropriate use of a grid and AEC system, high‐speed film‐screen, optimum exposure parameters, and total filtration. Replacement of old equipment and training radiography technicians are also essential. Regular inspection of radiological centers and implementation of QA programs will lead to lower patient doses and lower costs for medical health services.

## ACKNOWLEDGMENTS

We appreciate the efforts of all radiographers and specialists who helped us in this study, particularly Mr. Bayani. This paper was jointly conducted by Zahedan and Mashhad University of Medical Sciences, contract no. 89115.

## Supporting information

Supplementary Material

Supplementary Material

Supplementary Material
