# Supplementary material for: Local reference dose evaluation in conventional radiography examinations in Iran
Source: J Appl Clin Med Phys. 2014 Mar 6;15(2):303–10. doi: 10.1120/jacmp.v15i2.4550 (PMC5875487; doi:10.1120/jacmp.v15i2.4550)
Supplement: Supplementary file 1 — Supplementary Material [file ACM2-15-303-s001.doc]

Local reference dose evaluation in conventional radiography examinations in Iran

**M Shirin Shandiz[[1]](#footnote-2), 2, M.T Bahreyni Toosi^3,a^, S Farsi^1^, Kh Yaghobi^4^**

*Department of Medical Physics^1^, Zahedan University of Medical Sciences, Zahedan, Iran*

*Department of Medical Physics and Biomedical Engineering^2^, Tehran University of Medical Sciences, Tehran, Iran*

*Medical Physics Research Centre, Faculty of Medicine^3^, Mashhad University of Medical Sciences, Mashhad, Iran*

*Department of Occupational Health^4^, IRAN University of Medical Sciences, Tehran, IRAN,*

*bahreynimt@mums.ac.ir*

1. ^a^ Corresponding author: M.T Bahreyni Toosi, Professor of Medical Physics, Mashhad University of Medical Sciences, Mashhad, Iran.

   Fax: +98 511 8517505, email: bahreynimt@mums.ac.ir [↑](#footnote-ref-2)
