# Supplementary material for: Local reference dose evaluation in conventional radiography examinations in Iran
Source: J Appl Clin Med Phys. 2014 Mar 6;15(2):303–10. doi: 10.1120/jacmp.v15i2.4550 (PMC5875487; doi:10.1120/jacmp.v15i2.4550)
Supplement: Supplementary file 2 — Supplementary Material [file ACM2-15-303-s002.pdf]

EnglishEdit.ir

Certification Letter

TO WHOM MAY IT CONCERN:

This letter is to certify that this organization employs only native English-speaking citizens of English-speaking countries as editors of academic articles. EnglishEdit.ir certifies that the text of the article mentioned below has been edited by a native English speaker with appropriate experience and qualifications.

The substantive content of the article mentioned below remains the full responsibility of the author/authors:

Title of Article:

LOCAL REFERENCE DOSE EVALUATION IN CONVENTIONAL RADIOGRAPHY EXAMINATIONS IN IRAN

Author (s):

MAHDI SHANDIZ

Best Regards,

B. Radanpour
Radan English Edit
Head of Institute

Radan English Edit
www.englishedit.ir
info@englishedit.ir
No. 3, Sadaf, Motaheri, Darya , Sharak Gharb, Tehran, Iran.
Tel: +98 -21-22362401
