# Supplementary material for: Local reference dose evaluation in conventional radiography examinations in Iran
Source: J Appl Clin Med Phys. 2014 Mar 6;15(2):303–10. doi: 10.1120/jacmp.v15i2.4550 (PMC5875487; doi:10.1120/jacmp.v15i2.4550)
Supplement: Supplementary file 3 — Supplementary Material [file ACM2-15-303-s003.doc]

Local Reference Dose Evaluation in Conventional Radiography Examinations in Iran

Abstract

Introduction: This he goal of this study was to aims to establish local diagnostic reference Levels levels (LDRLs) for various conventional radiography examinations, in Sistan-Baluchestan province of Iran, using dose area product (DAP) measurements followed by a comparison with international dose levels.
Maals and Methods: 
was carried out at 8 radiography rooms in 6 public and 2 private health care centres. The study employed DAP, exposure, and demographic data (weight, age) for 1069 patients who presented for one of 11 routine radiography examinations, viz., chest (AP, PA, LAT), abdomen (AP), lumbar spine (AP, LAT), pelvis (AP), skull (AP/PA, LAT), and cervical spine (AP, LAT). The data was analyzed statistically and the minimum, median, mean, maximum, and third quartile DAP LDRL values were calculated.
Results: 
It was observed that LDRLs for chest PA (0.26 Gy.cm2) and chest LAT (0.66 Gy.cm2) projections were up to 136% and 120% higher, respectively, than their corresponding NRPB 2005 values. Other radiographic procedures had lower recommended reference doses compared with recently recommended national reference doses published in recent NRPB reports and other studies.
Discussion and Conclusion: Wide variations in DAP were observed for radiographic procedures between patients in different rooms and for different patients in the same room. These and other observations, such use of a grid with mid and low kVp techniques shows that the need to carry out quality assurance programs is critical in Iran. 

Keywords: radiography examination, dose area product, anti-scatter grid, exposure factors, radiation protection, DRL


1. Introduction

NCRP 160 states that medical radiation exposure of the United States population is almost half of total radiation exposure from natural and artificial resources. This is a result of the growing use of diagnostic imaging methods, particularly computed tomography(1); however, diagnostic radiography is still the most frequently used and accounts for most of the cumulative dose from diagnostic radiology methods(2).
The International Commission on Radiological Protection emphasizes three fundamental principles for protection in radiation diagnostic radiology. These are justification, protection optimization, and application of dose and risk limits.(3) The British National Radiological Protection Board (NRPB) emphasizes regular patient dose measurement in all radiological departments and diagnostic reference levels (DRLs) to optimize patient protection. To establish patient DRL for various radiography tests and raise public awareness about patient dose, it is useful to identify those centres associated with higher radiation doses. Following from this, adopting measures such as quality control of equipment can lead to a reduction in patient doses while maintaining image quality.(4)
The last 50 years of dosimetry in the United States have shown that the regular use of quality control programmes for diagnostic radiology equipment and the establishment of DRLs by the National Evaluation of X-ray Trends (NEXT) have played vital roles in reducing patient radiation doses.(5) For example, based on NEXT patient average entrance skin dose (ESD) and data such as reference dose levels, a 50%-70% reduction in average ESD was achieved for the years 1964 to 2004 for chest PA, abdomen AP, and lumbar-sacral spine AP radiography examinations.(5) Similar efforts have led to large reductions in patient doses in countries such as the UK. NRPB 2005 (HPA-RPD-029) reports that 20 years of regular patient dose monitoring has reduced DRLs by more than 50%.(6)
Radiological DRL values are explained in terms of ESD and dose area product (DAP).(6) DAP is the absorbed dose multiplied by the irradiated area. DAP is not only a quick and simple measurement, but also a very useful radiation dose qualifier. One important advantage is to mitigate the biological effects from both radiation dose and irradiated area of the body. DAP is also applicable for quality assurance and functional analysis of x-ray machines.(7-9)
The United Nations Scientific Committee on the Effects of Atomic Radiation (UNSCEAR 2000) reports that similar examinations in different countries and different districts of the same country may have different values stemming from cultural, scientific, and practical differences between regions. As a result, DRLs can be separately determined for a city, geographical area, or large health care centres as local diagnostic reference levels (LDRLs) while nationwide surveys establish national diagnostic reference levels (NDRLs).(10)
In a follow-up to previous studies, this study examines the patient dose information and LDRLs in Iran. LDRLs for common radiography procedures and with DAP measurements were recorded in Sistan-Baluchestan province, a comparatively disadvantaged and the less developed province in Iran.

II. Materials and Methods 

This study following guidelines established by NRPB 2005 (HPA-RPD-029). Since patient dosage depends on patient size, information was collected for adult patients over age 16 weighing 45 to 120 kg.(6) The average weight per patient in each radiography room was 70 ± 5 kg (similar to NRPB 2005).(6)
The study covered 8 radiography rooms in 6 public and 1 private health care centres. The rooms were randomly selected out of 33 functional rooms located in 27 state-owned and private radiography centres in Sistan-Baluchestan province. 
The province is comparatively disadvantaged and under-developed developed; at the time of the project, there was no digital radiography system in use.
 The radiographic devices used were one single-phase, 3 three-phase, and four high frequency devices. All radiographic devices used the film-screen system with a speed of 400 in all rooms. None of the devices had an automatic exposure control system.
Table 1 shows the results for the following diagnostic centres: Private Diagnostic Centre (room 1), Khatam Educational, Research and Treatment Centre of Zahedan (room 2), Private Diagnostic Centre (room 3), Khatam Educational and Treatment Centre of Iranshahr (room 4), Social Security Organization Hospital of Zahedan (room 5), Emam Ali Educational, Research and Treatment Centre of Zabol (room 6), Bu-Ali Educational and Treatment Centre of Zahedan (room 7), Emam Ali Educational and Treatment Centre of Chabahar (room 8).
A DAP meter (Gammex RMI, Model 840A, USA) calibrated according to the method proposed by the National Radiological Protection Board Protocol was used to obtain DAP values.(8) The system had a detector and a monitor. The detector was 14 cm x 14 cm and was installed under the beam collimator. It had a diagnostic energy range of 50 to 150 kVp and low absorption (less than 0.5 mm Al). 
After patient exposure, DAP values (mGy.cm2) and radiation time (ms) were transmitted via cable to the monitor. This and other radiographic data (kVp, mA, mAs, field dimension), patient information (age, height, weight), and radiography conditions (FFD, FSD, with or without grid, grid ratio) were recorded.
Average DAP values were calculated from the measurements according to NRPB standards for each room for the 11 conventional examinations considered in this study: chest (AP, PA, LAT), abdomen (AP), lumbar spine (AP, LAT), pelvis (AP), and skull (AP/PA, LAT). The third quartile DAP values were then calculated from the results for each the radiographic examination type and view and adopted as the LDRL in Sistan-Baluchestan province.(6)

III. Results

Average DAP values (Gy.cm2), total filtration (mm Al) and grid ratio for x-ray machines for the studied rooms are shown in Tables 1(a) and 2(a). Only one grid ratio was used for all rooms. 
Table 2 lists the radiological parameters (examination type, kVp, mAs) and the spread of the 1069 patients across the radiographic examinations. The age, gender, weight, and body mass index (BMI) of the patients are also presented in Table 2. 
Table 3 shows the statistical distribution of the average DAP by room and the minimum, maximum, mean, median, first quartile, third quartile, and maximum-to-minimum ratio for the 11 radiographic procedures. Statistical distributions for all radiographs were obtained; the chest AP had the minimum sample size in this study. 
Table 4 shows the average dose (Gy.cm2) for other studies for comparison purposes. Studies conducted by Bahreyni-Toosi et al. in 2006 in the city of Mashhad and in 2011 in the city of Sabzovar are the only studies in Iran that used DAP. Table presents results for NRPB 2000 (W14) and NRPB 2005 (HPA-RPD-029) for average dose and UNSCEAR 2000 recorded for nationwide patient dose evaluations conducted in Germany, New Zealand, and Finland. Also included are the results of a study by Bidemi et al, conducted on a limited scale in 4 hospitals in Nigeria.
Table 5 evaluates routine kVp and mAs for different types of radiographic examinations from different locations. Since there is a shortage of DAP studies, a review of studies were ESD was measured by Kim et al. (2007) in South Korea, Bahraini et al. (2008) in Tehran, and Sunavan et al. in India (2010) was used to obtain comparable data. 
Table 6 shows DRLs for different types of radiographic examinations in Sistan-Baluchestan province and recent NRPB reports.

IV. Discussion and Conclusion 

Tables 1, 2, and 3 show a wide range of DAP values and exposure parameters for similar radiographic procedures. These variations were observed in the same rooms, for a specific procedure for different patients and in different rooms for similar procedures. For instance, the maximum-to-minimum ratio of DAP for individual patients varied from 8 for chest AP to 70 for skull AP/PA. Such wide variations have also been reported elsewhere in diagnostic radiography practice(11-13) and suggest that doses can be reduced without loss in image quality. 
Table 4 shows that the average dose incurred by patients following chest AP, PA, and LAT projections are higher than corresponding values presented in NRPB 2000 (W14) and 2005 (HPA-RPD-029). At the same time, the patient dose in the present study for these examinations are smaller than analogous figures acquired by Bahreyni-Toosi et al. (2006; 2011) and some obtained by UNSCEAR 2000 in New Zealand and Finland. A comparison of other studies with the average doses from the present study shows they were lower than all the other international reports recorded in Table 4, but higher than Bahreyni-Toosi et al. (2011) in Sabzevar that used a similar film screen speed.(6,9,10,14-16) 
NRPB 2000 recommends the use of grids for high kVp (110–150 kVp) and not using grids with low and mid kVp ranges (60–90 kVp). Nevertheless, radiography technicians in this study often used grids for chest PA radiographies (Tables 1 and 2) in the low and mid kVp ranges (50-87 kVp).(9) Table 5 indicates that the use of grids with low and mid kVp is the main reason for higher mAs and DAP doses for chest radiographies than recommended in NRPB reports. This hypothesis, however, is not sufficient to explain DAP values for other radiographic examinations. It seems that DAP doses that are higher (e.g., chest radiographies) and lower (other radiographic procedures) than corresponding values presented in other reports (Table 5) may be a result of poor radiographic techniques in Sistan-Baluchestan province health care centres.(6,9,10,14-19) As Table 4 and 5 show, this weakness was also seen in Bahreyni-Toosi et al. in Tehran, Mashhad, and Sabzovar,(14,15,19) There is a clear need to develop QA programs in Iran. 
To achieve the ideal images using a grid, the mAs and DAP values should be increased in accordance with the grid factor. Unlike the law, In some radiography examinations (e.g. abdomen (AP), lumbar spine (AP, LAT), skull AP/PA), the exposure parameters and DAP values have not been approved for the grid factor and were not sufficiently increased (Tables 2, 4, and 5).(20) It was also determined that image quality was inadequate in this province. Future study should include an examination of image quality achieved with low and mid kVp examinations conducted with grids.
Table 6 shows similar to average doses for chest PA and LAT. The third quartile DAP values for Sistan-Baluchestan province that were recommended as the LDRL are higher than NRPB 2005 (HPA-RPD-029) by up to 136% and 120%, respectively.(6) There was insufficient data for the chest AP (Table 3; 4 centres and rooms, 21 patients), therefore, a recommended LDRL was not developed for this procedure.
Since Sistan-Baluchistan is underdeveloped, all centres in this study used only film screens. Only 55% of centres in the NRPB 2005 (HPA-RPD-024) used film screens; the rest were equipped with CR and DR image receptors. Most x-ray machines were equipped with an AEC system, which may affect the magnitude of DAP and, consequently, DRLs. The radiography technology and film-screen speed applied in this study (speed class 400) were similar to NRPB 2000 (W14). About 98% of radiography devices use a film-screen system with an average film screen speed of 390. 
The LDRL for chest PA radiography is 117% higher than the corresponding NRPB 2000 value, but recommended local reference doses for abdomen AP, lumbar spine AP, lumbar spine LAT, and pelvis AP for Sistan-Baluchestan province are lower than those in NRPB 2000 by 45%, 36%, 34%, and 45%, respectively (Table 6).(6,9) These findings confirm that radiographic techniques in Sistan-Baluchestan province health care centres are inadequate.
DRLs for cervical spine AP and LAT using DAP measurements were not reported in NRPB and other studies. Table 6 shows that the first DRLs for these are 0.16 and 0.25 Gy.cm2, respectively. 
Average BMI was 25kg/m2 and average weight was 66 kg (Table 2); this average weight is less than the 70 kg mean weight in NRPB 2000 and 2005. BMI was not considered in the recent NRPB reports. This factor is a function of weight and height and patients with larger mass require higher technical parameters (kVp and mAs). Average BMI is higher in Sistan-Baluchestan than analogous values in European countries; larger BMI values could lead to higher DRLs.(6,9)
In 2005, more than 6 million chest radiographies were performed, accounting for 30% of the total radiography examinations nationwide. The number of chest x-rays was larger and DRL for chest x-rays were higher in this study than for similar studies. The wide variation in DAP values observed for similar radiographic procedures among patients in different rooms and for different patients in the same room are all convincing reasons for a comprehensive QA program. 
Such a QA program should implement measures such as using the smallest possible radiation field, appropriate use of a grid and AEC system, high speed film-screen, optimum exposure parameters and total filtration. Replacement of old equipment and training radiography technicians are also essential. 
Regular inspection of radiological centres and implementation of QA programs will lead to lower patient doses and lower costs for medical health services.

Acknowledgements: We appreciate the efforts of all the radiographers and specialists who helped us in this study, particularly Mr. Bayani. This paper was jointly conducted by Zahedan and Mashhad Medical Science Universities, No. 89115.


Table 1(a). DAP (mean ± SD) for Gy.cm2 for radiographies from selected radiography rooms 
Table 1(b). DAP (mean ± SD) for Gy.cm2 for radiographies from selected radiography rooms. 
Table 2. Average patient characteristics and exposure parameters.
Table 3. Statistical distribution of average DAP per room.
Table 4. Average DAP for present study versus other studies.
Note: a, b, c, d, e, f = values are reported together. 
Table 5. Conventional average radiation parameters (kVp, mAs) from different studies.
Table 6. DRLs from present survey versus recommended national reference doses from recent NRPB reports.
